# Tumor associated macrophages polarization dictates the efficacy of BCG instillation in non-muscle invasive urothelial bladder cancer

**DOI:** 10.1186/1756-9966-32-87

**Published:** 2013-11-05

**Authors:** Francesca Suriano, Daniele Santini, Giuseppe Perrone, Michela Amato, Bruno Vincenzi, Giuseppe Tonini, Andrea Onetti Muda, Sara Boggia, Maurizio Buscarini, Francesco Pantano

**Affiliations:** 1 Department of Urology, Campus Bio-Medico, University of Rome, Via Spilimbergo 10, int 35, 00182 Rome, Italy; 2 Department of Oncology, Campus Bio-Medico, University of Rome, Via Alvaro del Portillo 200, 00128 Rome, Italy; 3 Department of Anatomical Pathology, Campus Bio-Medico, University of Rome, Via Alvaro del Portillo 200, 00128 Rome, Italy; 4 Sant’Andrea Hospital, Rome, Italy

**Keywords:** Bladder cancer, Endovescical instillation, Tumor-associated macrophages, Macrophages polarization, Prognosis

## Abstract

**Background:**

To evaluate the prognostic role of TAMs in patients affected by non-muscle invasive bladder cancer (NMIBC), undergone Trans Urethral Resection of Bladder (TURB) and Bacillus Calmette-Guerin (BCG) therapy.

**Methods:**

Data from 40 patients (36 men, 4 women), mean age 69 years (40-83 years), treated for NMIBC with TURB and BCG instillation were collected. Two different groups were considered: group with and group without bladder cancer recurrence. Correlations between immunofluorescence measured Mtot, M1 and M2 infiltration and clinicopathological parameters were evaluated using Spearman and Mann–Whitney methods. The recurrence-free survival rate was calculated using the Kaplan-Meier method.

**Results:**

CD68 positive cells (Mtot) were observed in all specimens tested. High Mtot, M1 and M2 infiltration was observed in patients with disease recurrence, even before endovescical BCG instillation. Significant value for M2 infiltration (*p* = 0,042) was found calculating significativity between two group medians before BCG therapy. *p* = 0,072 and *p* = 0,180 were observed correlating median of Mtot and M1 between two groups of patients respectively. Values of *p* = 0,44, *p* = 0,23 and *p* = 0,64 from correlation between DFS and Mtot, M1 and M2 median in patients before endovescical BCG instillation, were calculated respectively. Comparing DFS and Mtot, M1 and M2 median in patients group after endovescical BCG instillation significant values were obtained (*p* = 0,020; *p* = 0,02; and *p* = 0,029 respectively).

**Conclusions:**

M2 tumor infiltration could be a prognostic value of recurrence in patients with NMIBC.

## Background

Urothelial bladder cancer is the second cancer for incidence of urinary tract. In 2008, 90.900 new cases in Europe (86.300 males and 4.600 females) have been reported. Bladder cancer is responsible of 4.1% cancer-correlated death in men and 1.8% in women [1].

75% of urothelial bladder cancer are non-muscle invasive (NMIBC) at diagnosis [2]. Standard therapy for NMIBC includes trans-urethral resection of tumor, followed by endovescical instillation of chemo- / immuno-therapy for high grade disease [3-5]. *Mycobacterium bovis* (Bacillus Calmette Guerin–BCG) has been established as the most effective adjuvant treatment for decreasing recurrence and tumor progression risk. Since its first use in 1976 [6] major efforts have been directed to understand the mechanism of BCG mediating anti-bladder cancer immunity. Despite its clinical benefit the mechanism underlying the antitumor activity of intravescical BCG instillation has not been clarified. However, it has been reported that intravescical BCG provokes an inflammation involving the contribution of various immune cells including cells associated with the innate immune response. Data from Ayari et al suggested that patients with high level of infiltration by TAMs in the cancer area don’t respond as well to BCG immunotherapy [7].

Tumor-associated macrophages (TAMs) represent a substantial fraction of the growing tumor mass and are associated with poor prognosis in several human cancers [8]. TAMs exist in two different polarizations state classified as M1 and M2. M1 macrophages show a protective role in tumor-genesis activating tumor-killing mechanisms and antagonizing the activities of M2.

M2 macrophages are clearly involved in suppression of adaptive tumour-specific immune responses and in promotion of tumour growth, invasion, stroma remodelling and angiogenesis [9-13].

Considering the rationale of BCG use, we hypothesized that endovescical instillation efficacy could be modulated according to TAM polarization and conversely macrophage could be influenced by BCG itself.

## Material and methods

A total of 40 patients (36 males and 4 females), mean age 69 years (40-83 years), diagnosed with non-muscle invasive bladder cancer (NMIBC) at our institution (*Campus Bio-Medico, University of Rome*) from 1999 to 2011 were selected randomly for study. Between them, 23 patients had not recurrence at follow-up versus 17 patients with bladder cancer recurrence.

Diagnosis of bladder cancer was made by histological examination of specimens obtained by transurethral bladder biopsy. Histological specimens were fixed in 10% neutral buffered formalin and routinely processed for paraffin embedding. Serial 5 μm sections were cut, stained with hematoxylin and reviewed by a pathologist.

All patients underwent same intravescical BCG regimens (80 mg Immucyst/80 ml Salin solution 0.9%). After initial therapy, patients were followed with periodic cystoscopy, urine cytology and Uro-TC.

**Figure 1:**
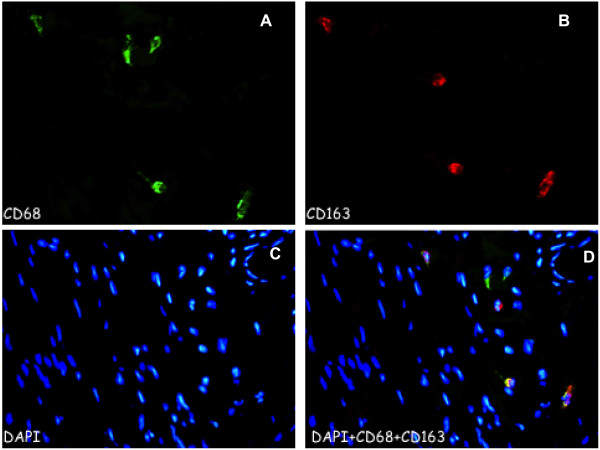
**CD68/CD163 expression in M2 macrophage in bladder cancer. A)** CD68 (green), shows nucleated cells positive staining for CD68; **B)** CD163 (red 2), shows CD163 staining in macrophage phenotype; **C)** DAPI, shows the cell nuclei marked with DAPI; **D)** merged image of DAPI, CD68 and CD163 showing a number of macrophages with positive staining for the phenotype marker M2. Original magnification × 400.

**Figure 2:**
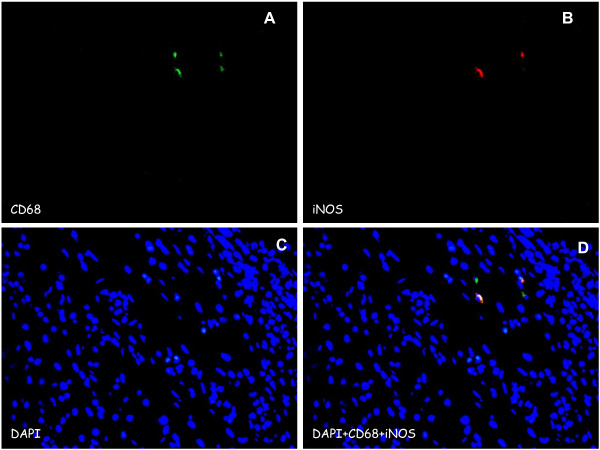
**CD68/iNOS expression in M1 macrophage in bladder cancer. A)** CD68 (green), shows nucleated cells positive staining for CD68; **B)** iNOS (red), shows iNOS staining in macrophage phenotype 1; **C)** DAPI, shows the cell nuclei marked with DAPI; **D)** merged image of DAPI, CD68 and iNOS showing a number of macrophages with positive staining for the phenotype marker M1. Original magnification × 400.

We evaluated two consecutive histological sections (before and after intravescical BCG instillations) by Immunoflorescence. Histologic reviewers were blinded to recurrence outcomes. TAMs were labeled using CD68 monoclonal antibody (monoclonal mouse clone PG-M1), Ab anti-iNOS (Rabbit mAb) and Ab anti-CD163 (Rabbit mAb; 1:200). DAPI was used for detection of nucleate cells. Cells positive for CD68 were considered whole macrophage population (Mtot); cells positive for CD68 and CD163 were considered M2 population and those positive for CD68 and iNOS were considered M1 population (Figures 1 and 2).

For systematic counting 5 high power fields were chosen randomly under a microscope (Eclipse 80i Nikon microscope, Tokyo, Japan) at 400× magnification.

In order to assess whether there is any value of the macrophage density of M1 and M2 in predicting prognosis, the median value of the macrophage density of two populations was used as a cut-off point to dichotomize the 40 patients into a group with a macrophage density above or below the median value.

Statistical analysis was performed using SPSS software (vers. 17). Correlations between immunofluorescence measured Mtot, M1 and M2 infiltration and clinical-pathological parameters were evaluate using Spearman and Mann–Whitney methods. The recurrence-free survival rate was calculated using the Kaplan-Meier method.

## Results

**Figure 3:**
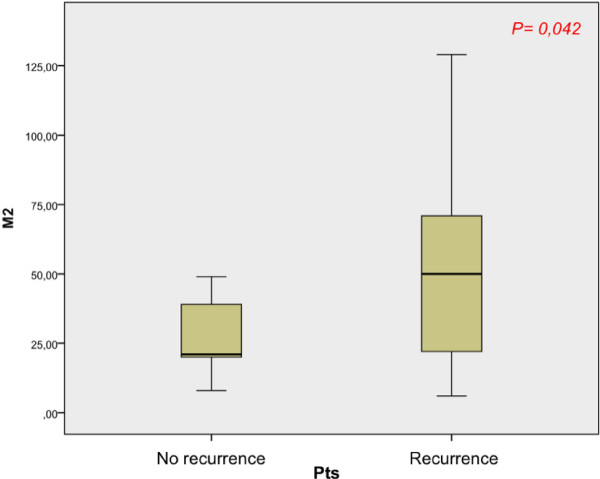
Correlation between M2 median of two groups of patients (recurrence and no recurrence).

**Figure 4:**
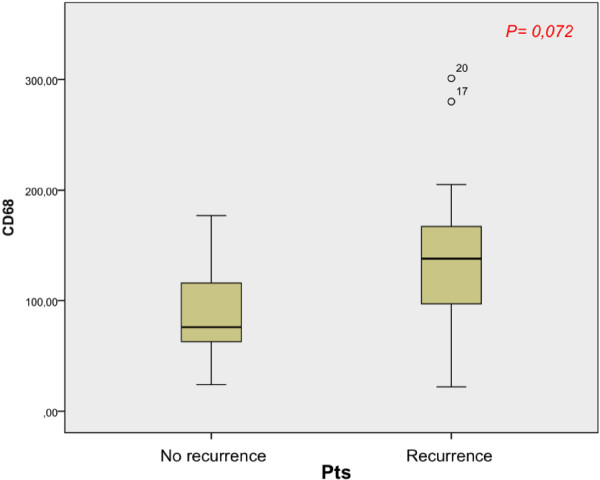
Correlation between Mtot median of two groups of patients (recurrence and no recurrence).

**Figure 5:**
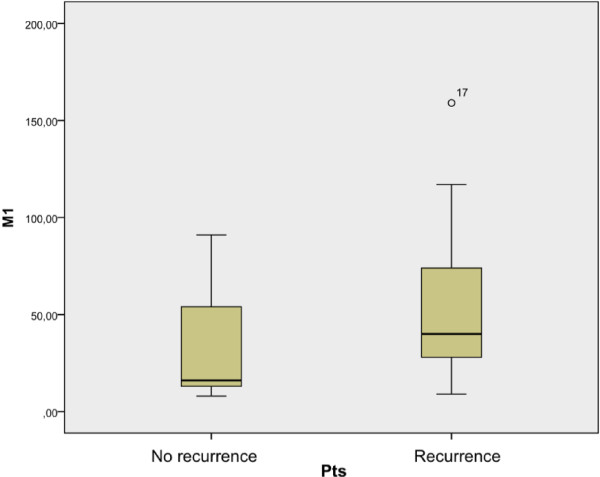
Correlation between M1 median of two groups of patients (recurrence and no recurrence).

CD68 positive cells (Mtot) were observed in all specimens tested. Considering two patient populations (recurrence and no-recurrence groups) we found a different M1 and M2 infiltration (Tables 1 and 2). We observed a higher Mtot, M1 and M2 infiltration in patients with disease recurrence, even before endovescical BCG instillation. Calculating significativity between two groups median before BCG therapy, we found a significant value for M2 infiltration (p = 0,042) (Figure 3). Instead, there were not significant values correlating median of Mtot and M1 between two groups of patients (p = 0,072 and p = 0,180 respectively) (Figures 4 and 5).

**Table 1:** Patients without recurrence

***Before BCG***	***After BCG***
***CD68 (median: 36, IQR1-3: 30-47)***	***CD68 (median: 20, IQR1-3: 13-25)***
***CD68/CD163 (median:21, IQR1-3: 20-39)***	***CD68/CD163 (median:14, IQR1-3: 10-24)***
***CD68/INOS (median: 16, IQR1-3: 13-54)***	***CD68/INOS (median: 17, IQR1-3: 9-22)***

**Table 2:** Patients with recurrence

***Before BCG***	***After BCG***
***CD68 (median:59, IQR1-3:44-92)***	***CD68 (median: 53, IQR1-3:33-101)***
***CD68/CD163 (median:50, IQR1-3:22-71)***	***CD68/CD163 (median:37, IQR1-3:21-77)***
***CD68/INOS (median:40, IQR1-3:28-74)***	***CD68/INOS (median: 34, IQR1-3: 24-66)***

**Figure 6:**
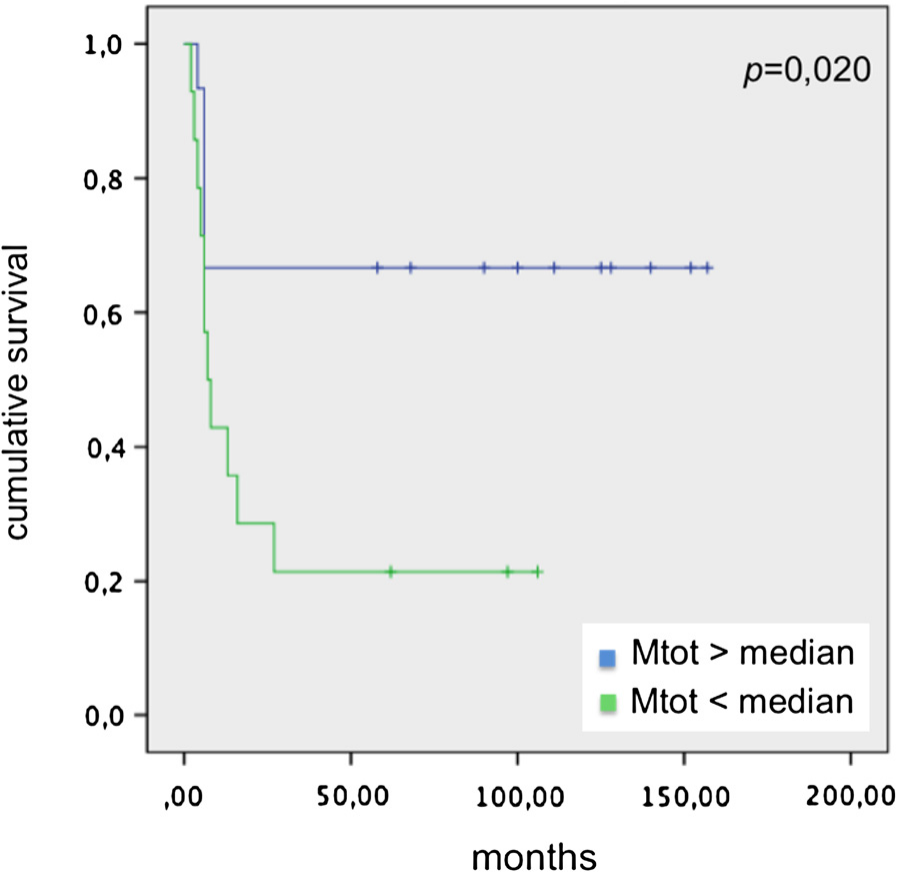
DFS and Mtot median in patients underwent BCG instillation.

**Figure 7:**
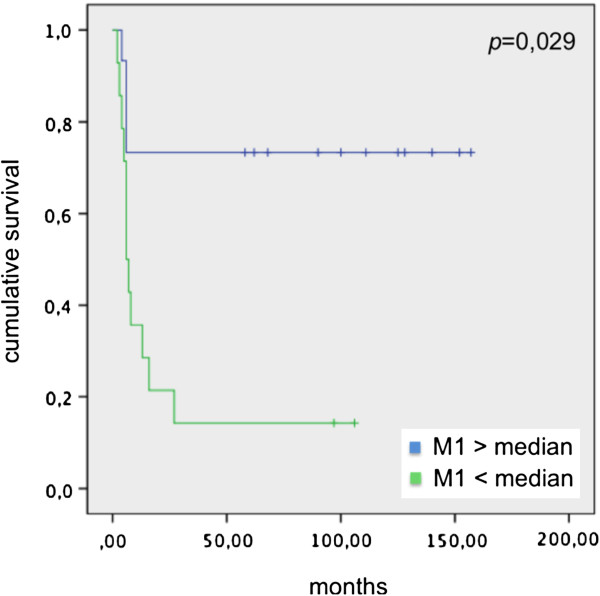
DFS and M1 median in patients underwent BCG instillation.

**Figure 8:**
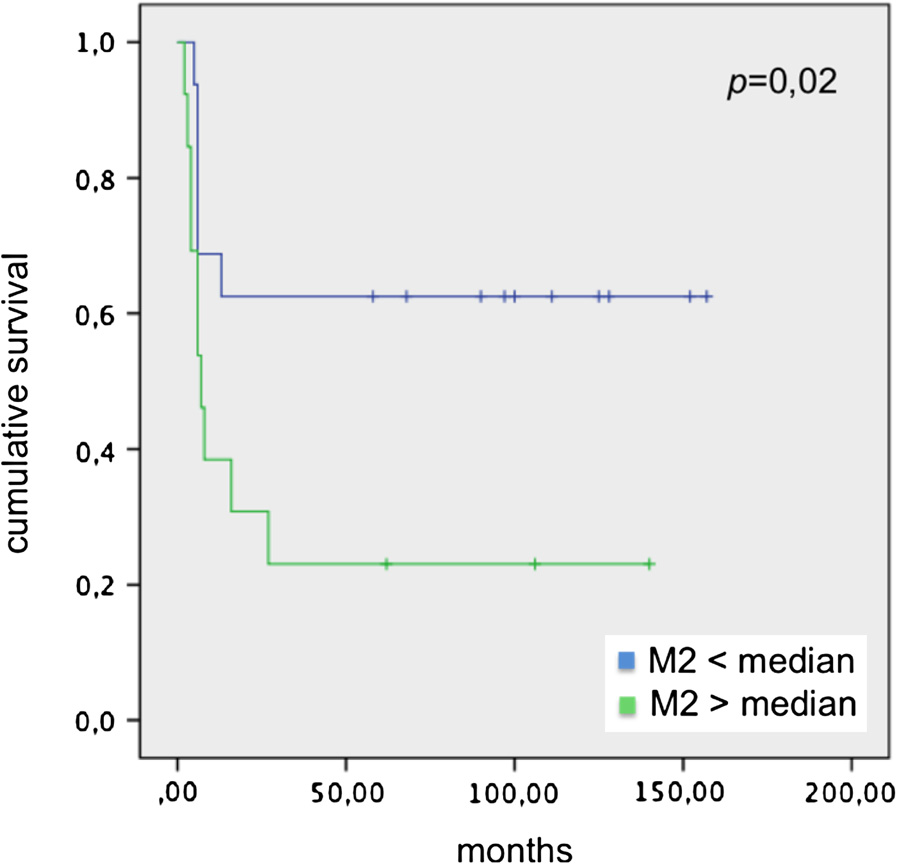
DFS and M2 median in patients underwent BCG instillation.

Correlating disease-free survival (DFS) and Mtot, M1 and M2 median in patients before endovescical BCG instillation, we didn’t observe significant values. *p* = 0,44 from correlation between DFS and Mtot median, *p* = 0,23 from correlation between DFS and M1 median, *p* = 0,64 from correlation between DFS and M2 median were calculated. On the contrary, significant values comparing DFS and Mtot, M1 and M2 median in patients group after endovescical BCG instillation (*p* = 0,020; *p* = 0,02; and *p* = 0,029 respectively) were present (Figures 6, 7 and 8).

## Discussion

Bladder cancer is one of the most widespread cancers afflicting men and women, and its incidence grows exponentially each year. Early studies reported that the macrophages increase in bladder cancer is associated with high survival and invasive capacity [14]. Activated macrophages promote tumor-genesis through the expression of growth factors and matrix proteases, promotion of angiogenesis and suppression of anti-tumoral immune response [14,15]. As Dufresne et al described in their study [16], pro-inflammatory M1 should suppress tumor growth; instead anti-inflammatory M2, via production of IL-10 and other soluble factors, suppress the anti-tumoral effects of M1.

In many human neoplasms, including lung, breast, cervix, ovary and pancreas cancers, the presence of extensive TAM infiltrate correlates with poor prognosis. In other tumors, including brain and prostate cancer, there is conflicting evidence regarding the role of macrophages in survival outcomes [17-21]. The basis for these conflicting data may be explained considering that in these studies tumor-associated macrophages were detected only by the immunohistochemical analysis of CD68+ cells. In fact both M1 and M2 phenotypes share the expression for CD68, therefore the use of CD68 alone might not represent a reliable marker in evaluating the real impact of the two subtypes.

The role of TAM in non-muscle invasive bladder cancer was previously investigated by Ayary et al finding a role of this infiltrate in modulating BCG efficacy [7]. Anyway this work did not take into account the real role of the two opposite macrophage population.

In our study we used double-staining for CD68/NOS2 as markers for M1 macrophages and CD68/CD163 as markers for M2 macrophages to be in accordance with the most part of previously published studies that performed a phenotypic characterization of macrophages polarization [17,20-27].

The haemoglobin scavenger receptor, CD 163, is expressed almost exclusively on macrophages and monocytes, and it is strongly upregulated by anti-inflammatory cytokines, important for M2 polarization. Conversely, macrophages M1 polarized by exposure to interferon (IFN)-γ or LPS up-regulate inducible nitric oxide synthase (iNOS) to convert into nitric oxide (NO) that combining with oxygen radicals leads to the formation of cytotoxic peroxynitrite. These markers are not absolutely specific, for example CD68 has been found in immature CD1a-positive dendritic cells.

CD163 is also expressed in some dendritic cells, and iNOS is expressed by endothelial cells as well as by arterial wall smooth muscle cells. For these reasons we have given particular attention to cell morphology in order to minimize potential bias [20-23,28-31].

## Conclusion

In this study we investigated the role of tumor-infiltrating macrophages in non-muscle invasive bladder cancer. In particular, we obtained more insight in clinical significance of macrophage polarization in predicting recurrence after BCG instillation. Our findings support the idea that a sustained M2 infiltration in tumor microenvironment could significantly limit the efficacy of BCG suggesting the need of a well planned therapeutical strategy in non-muscle invasive bladder cancer patients.

## Competing interests

The authors declare that they have no competing interests.

## Authors’ contribution

FS and FP were the main authors of the manuscript; SB and FP collected and studied the bibliography; DS, MB, GT, AOM and BV participated in the sequence alignment and drafted the manuscript; FS corrected the language form; MA and GP carried out immunohistochemical studies; FS drafted the article and revised it critically for important intellectual content. All authors read and approved the final manuscript.
